# Dietary polysaccharide-rich extract from *Eucheuma cottonii* modulates the inflammatory response and suppresses colonic injury on dextran sulfate sodium-induced colitis in mice

**DOI:** 10.1371/journal.pone.0205252

**Published:** 2018-10-05

**Authors:** Sabri Sudirman, Yuan-Hua Hsu, Jia-Ling He, Zwe-Ling Kong

**Affiliations:** Department of Food Science; National Taiwan Ocean University, Keelung City, Taiwan; Toho University Graduate School of Medicine, JAPAN

## Abstract

Inflammatory bowel disease (IBD) is a known medical burden in most developed countries and a significant cause of morbidity. The IBD label includes Crohn’s disease (CD) and ulcerative colitis (UC). Pharmacological and surgical intervention are the two main management approaches for IBD. Some drugs have been developed for IBD therapy, but accessibility is limited due to high costs. Furthermore, these agents have demonstrated inactivity over long-term treatment courses. Therefore, an urgent need is present for new treatment options that are safe, able to sustain clinical remission, and improve mucosal gut healing. Seaweed has received much attention in the pharmacological field owing to its various biomedical properties, including the prolongation of blood clotting time, as well as antitumor, anti-inflammation, and antioxidant effects. This study therefore aimed to examine the effects of a dietary polysaccharide-rich extract obtained from *Eucheuma cottonii* (EC) on a model of colitis. Colitis was induced in male BALB/c mice by the administration of 2.5% (w/v) dextran sulfate sodium (DSS) for 7 days. DSS-induced mice were treated with either one of three different doses of EC extracts (0.35, 0.70, and 1.75 g/kg body weight) or curcumin as a positive control (0.10 g/kg). Mice were sacrificed post-treatment and blood samples were collected. The disease activity index (DAI) and inflammatory cytokine levels (tumor necrosis factor (TNF)-α, interleukin (IL)-1β, IL-6, IL-10) were measured. After treatment for 7 days, EC extract administration protected against weight loss and decreased the colon weight per length ratio. EC extract administration also decreased pro-inflammatory cytokine expression, increased IL-10 levels, and reduced colonic damage. Therefore, a dietary polysaccharide-rich extract from *E*. *cottonii* reduced DSS-induced bowel inflammation, thereby becoming a promising candidate for the treatment of colitis.

## Introduction

Inflammatory bowel disease (IBD), a designation that includes Crohn’s disease (CD) and ulcerative colitis (UC), is a known medical burden in most developed countries [1, 2]. UC is characterized by intestinal inflammation and often results in diarrhea, bloody mucus, weight loss, and colon shortening [1, 3]. Since the mid-twentieth century, IBD incidence has increased in the Western world (e.g., North America, Europe, New Zealand, and Australia) [4], but only recently have newly industrialized countries in Asia, the Middle East, and South America documented the emergence of IBD [5]. Furthermore, increasing IBD prevalence has also been observed in newly industrialized countries with large populations and rapid urbanization and westernization such as India and China. As such, IBD can be categorized as a global disease [4, 5].

Currently, pharmacological and surgical intervention are the two main treatment approaches for IBD [6]. Traditional therapeutic agents such as azathioprine, 6-mercaptopurine, and antibiotics are becoming more important in steroid-resistant and steroid-dependent patients [7]. Drugs such as corticosteroids, aminosalicylates, and immunosuppressants aim to decrease inflammation, but show limited efficacy for long-term remission and present significant side effects [8]. As such, natural products, including those with marine origins, have been investigated in order to identify potential candidates for the improvement of IBD clinical symptoms [9].

Seaweeds, also known as marine algae, are now considered as potential sources for new IBD treatments. Some seaweeds can be used for the management or treatment of IBD, including *Caulerpa mexicana* [1], a sulfated polysaccharide from *Hypnea musciformis* [10], and fucoidan extract from *Fucus vesicolosus* [6]. These seaweeds have many bioactive compounds, such as polysaccharides, terpenes, and flavonoids, which have been documented to possess pharmacological activities including antitumor, antiprotozoal, antiviral, antioxidant, anti-nociceptive, anti-inflammatory, and anticoagulant effects [1, 11–13]. Seaweeds contain large amounts of polysaccharides, but the majority of them are not digested by humans due to the absence of the required enzymes in the gastrointestinal tract. Therefore, these can be regarded as dietary fibers, resulting in seaweed being classified as having high levels of dietary fibers (33–75%) [14, 15]. The proportion of dietary fiber is particularly rich in the soluble fraction (50–85% of the total dietary fraction), which in red seaweeds is mostly composed of sulfated galactans such as carrageenan and agar [15]. Dietary fiber has been used to treat colitis [16, 17] and modulate the gut microbiota [18, 19].

*Eucheuma cottonii* is a red seaweed previously reported to demonstrate antioxidant, anticoagulant, anti-tumor, and anti-inflammation properties [20–24]. *Eucheuma cottonii* is also known as *Kappaphycus alvarezii* (KA) or the “sea-bird nest” [25, 26]. In addition, studies have reported that extracts produced from this seaweed can slow tumor cell growth rate [27], promote wound healing [28], and upregulate cancer cell apoptosis [29]. Moreover, KA extracts have been shown to improve cardiovascular, liver, and metabolic parameters in obese rat models [30] and present anti-diabetic effects in streptozotocin-induced type 2 diabetic mice [31]. However, the effects of a dietary polysaccharide-rich extract from *Eucheuma cottonii* in a murine model of colitis has not been reported.

Various chemical agents can be used to induce colitis in rodent models, including dextran sodium sulfate (DSS), trinitrobenzene sulfonic acid (TNBS), oxazolone, acetic acid, carrageenan, indomethacin (a non-steroidal anti-inflammatory drug [NSAID]), and peptidoglycan-polysaccharides [32]. Acute or chronic colonic inflammation can be induced by DSS administration via drinking water, with its effects depending on dosage and duration [33]. The DSS colitis model is popular owing to its controllability, reproducibility, simplicity, and rapidity [34] and it has been confirmed to represent colitis both biochemically and morphologically [35]. This study therefore aimed to examine the suppressive effects of a dietary polysaccharide-rich extract from *Euchuema cottonii* against DSS-induced colitis in mice.

## Materials and methods

### Materials

*Eucheuma cottonii* (EC) was provided by a seaweed farm in Sabah (Famous Alpine Sdn. Bhd, Sabah, Malaysia). Curcumin was purchased from Nacalai Tesque, Inc. (Kyoto, Japan). Dextran sulfate sodium (DSS, MW ~40.000 Da) was purchased from Tokyo Chemical Industry (Tokyo, Japan). Tumor necrosis factor (TNF)-α (Cat. No. ARG80206), interleukin (IL)-6 (Cat. No. ARG80199), IL-1β (Cat. No. ARG80196), and IL-10 (Cat. No. ARG80200) ELISA kits were purchased from Arigo Biolaboratories Corporation (Hsinchu, Taiwan). Formaldehyde solution (4%) was purchased from Avantor Performance Materials, Inc. (Pennsylvania, U.S.A.). Dulbecco’s modified Eagle’s medium-low glucose (DMEM-L) and Dulbecco’s phosphate buffered saline (PBS) powders were purchased from Sigma Aldrich (Missouri, U.S.A). Antibiotic-Antimycotic (15240–062) was purchased from Gibco Life Technologies (Massachusetts, U.S.A.).

### Seaweed extraction and characterization

#### Seaweed extraction

Seaweed extraction was performed according to previously used methods [36]. Briefly, 40 g of *Eucheuma cottonii* dried powder was placed in an Erlenmeyer flask and macerated with 200 mL of 70% ethanol. Extraction was performed at 50 °C for 3 h with stirring by a magnetic stirrer. The solution was filtered to separate the liquid ethanol extract from residue, with residue taken for repeated extraction by adding fresh solvent under the same conditions as the first extraction. Three extractions were performed in total. The ethanol extracts were then combined with the solvent then evaporated using a vacuum evaporator at 40 °C, resulting in a concentrated ethanol extract. Concentrated extract was transferred into fresh bottles and dried using a freeze dryer in order to obtain the final *E*. *cottonii* ethanol (EC) extract. The extraction yield (%) was calculated as extract weight (g) divided by dried seaweed powder weight (g) x 100%. The extraction yield was 17.79 ± 0.09%.

#### Seaweed extract characterization

EC extract proximate analysis was performed by following AOAC (2000) methods. Extract moisture content was determined in triplicate by drying at 105 °C in a hot-air oven to obtain a constant dry weight. Total nitrogen content was determined by Kjeldahl nitrogen analysis. The percentage of crude protein was estimated by multiplying total nitrogen content by a factor of 6.25 (AOAC method 981.10). Crude fat content was determined by extracting a dry ground sample for 6 h in a Soxhlet extractor with ethyl ether (AOAC method 991.36). Ash content was determined by complete incineration of the sample in a muffle furnace at 550 °C (AOAC method 930.05). Carbohydrate content was determined by the difference method.

Previously used methods were used to analyze 3,6-anhydro-galactose levels [37]. Briefly, resorcinol reagent was mixed with standards or samples in ice water for 3 min. Mixtures were then moved into a water bath and incubated at 80 °C for 10 min to allow for color development. After this, they were immediately transferred to a 96-well microplate to read absorbance at 520 nm. D-fructose (250 μmol/L) was used as a standard, with 55% of the solution an equimolar amount of 3,6-anhydro-α-D-galactopyranoside [38].

#### Cell viability assay

RAW 264.7 macrophages (RAW 264.7) were purchased from Bioresearch Collection and Research Center (BCRC), Taiwan and cultured in DMEM-L containing 1 g/L of glucose and *L*-glutamine and supplemented with 1% of antibiotic/antimycotic and 10% fetal bovine serum (FBS). Cells were maintained at 37 °C in a humidified atmosphere (5% CO_2_).

Cellular viability was performed by using a 3-[4,5-dimethylthiazol-2-yl]-2,5 diphenyl tetrazolium bromide (MTT)-based colorimetric assay [39]. Briefly, RAW 264.7 cells (1x10^5^ cells/well) were cultured in 96-well microplates with different concentrations of EC extract (125, 250, 500, or 1000 μg/mL) or in the absence of EC extract (control) for 24 h. Culture supernatant was then removed and cells were washed with phosphate buffer saline (PBS). After this, 100 μL of MTT solution (1 mg/mL) was added to each well and cells were incubated for 4 h at 37 °C. After incubation, cells were dissolved with 100 μL of dimethyl sulfoxide (DMSO) and shaken at room temperature in the dark for 15 min, after which absorbance was recorded at 570 nm.

### Animal models

Six-week-old male BALB/c mice were purchased from the National Laboratory Animal Center (Yilan, Taiwan). All mice were fed a standard chow-fed diet and water *ad libitum*. Mice were acclimatized for 1 week. Mice were housed 4 mice per cage in a room maintained at 25 ± 2 °C under a 12 h day/night cycle throughout experimentation. All procedures were carried out according to the Animal Protection Act (Act/APC) and the Experimental Animal Ethics Committee of the Council of Agriculture (CoA) of the Executive Yuan, Taiwan. The Institutional Animal Care and Use Committee (IACUC Approval No. 107003) of the National Taiwan Ocean University reviewed and approved all protocols.

**Fig 1:**
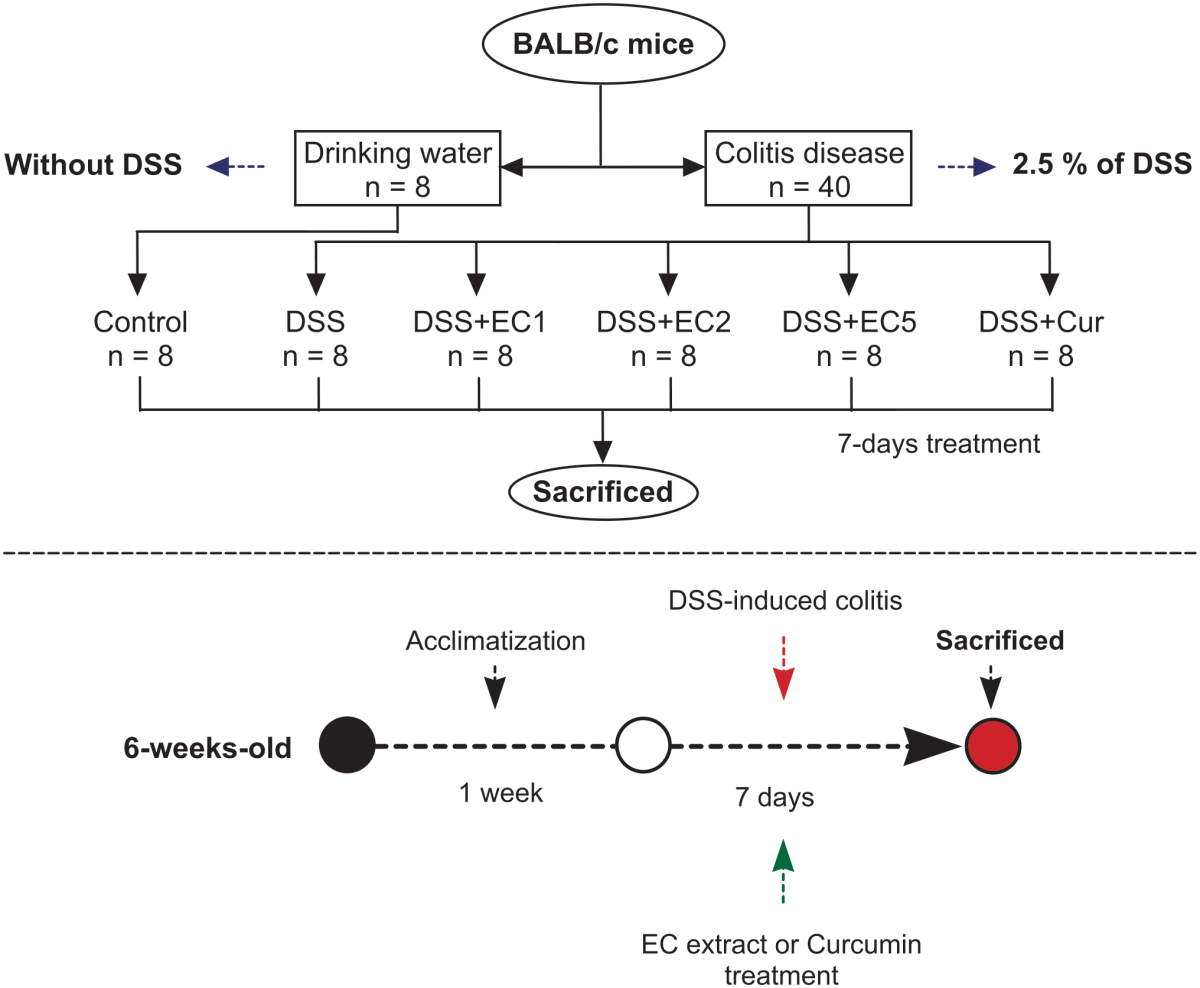
Flowchart of dextran sulfate sodium (DSS) induction of colitis in mice.

Acute colitis was established using a previously established method [35]. Briefly, mice were administered 2.5% (w/v) DSS in drinking water for 7 days [34]. Forty-eight mice were weighed prior to experimentation and then divided into six groups (8 mice per group), one which received no DSS administration (control) and five which did (DSS groups). DSS groups were then administered either varying dosages of EC extract by oral gavage (0.35 g/kg body weight, DSS+EC1; 0.70 g/kg, DSS+EC2; or 1.75 g/kg, DSS+EC5), curcumin (0.10 g/kg, DSS+Cur), or water (DSS) (Fig 1). Mice were examined daily for body weight, stool consistency, and the presence of fecal blood. EC extract and curcumin were orally delivered once per day of DSS treatment for 7 days. The mice were sacrificed on the 8^th^ day and fasted for 12 h prior to sacrifice. Mice were euthanized by CO_2_ exposure in an empty chamber. The colon weight and length were measured on the day of sacrifice. Serum and colon tissue were stored at -20 °C until further analysis.

### Disease activity index

Disease activity index (DAI) was measured by previous methods [35, 40, 41] and calculated based on the presence of fecal blood, stool consistency, and weight loss percentage. DAI values were calculated as [(weight loss score) + (stool consistency) + (rectal bleeding score)]/4 and scored on a 0–4 scale (Table 1).

**Table 1:** Disease activity index (DAI) scoring of DSS-induced colitis.

Score	Occult/gross bleeding	Weight loss (% of initial weight)	Stool consistency
0	None	< 1	Normal stools
1	Small spots of blood stool; dry anal region	1–5	Soft pellets not adhering to the anus
2	Large spots of blood in stool; blood appears through the anal orifice	5–10	Very soft pellets adhering to the anus
3	Deep red stool; blood spreads largely around the anus	10–15	Liquid stool in long streams; wet anus
4	Gross bleeding	> 15	Diarrhea

Table adapted from previous studies [35, 40, 41].

### Inflammatory cytokine level analysis

Blood serum was collected using a syringe and centrifuged for 15 min at 3,000 rpm and stored at -20 °C. The supernatant of homogenized colon tissue was prepared by weighing 100 mg of colon tissue, suspending it in 900 μL of cold PBS, and then homogenizing using a micro-tube homogenizer. Preparations were stored at -20°C, then thawed at room temperature prior to use. Homogenized tissue was centrifuged at 5,000 rpm for 15 min and the supernatant was collected for the cytokine analysis [42, 43]. Pro-inflammatory cytokines (TNF-α, IL-1β, and IL-6) and anti-inflammatory (IL-10) cytokines were detected using enzyme-linked immunosorbent assay (ELISA) kits via manufacturer’s protocols.

### Histopathology analysis

Upon sacrifice, mouse colons were collected and the distal colon was fixed in 4% neutral-buffered formaldehyde solution. Specimens were then embedded in paraffin after fixation and 5 μm-thick sections were cut and stained with hematoxylin and eosin (H&E) as described by previous methods [44] by Lie Pei Co., Ltd. Colonic histology was scored according to previous methods [45, 46]: Scores were defined as: 0, normal colonic mucosa; 1, loss of one-third of the crypts; 2, loss of two-thirds of the crypts; 3, lamina propria covered with a single layer of epithelial cells with mild inflammatory cell infiltration; and 4, erosions and marked inflammatory cell infiltration.

### Statistical analysis

All data were expressed as mean ± standard deviation (S.D.). The data were analyzed using Duncan’s multiple range test (P<0.05) using SPSS 22.0.

## Results

### EC extract chemical composition

Proximate analysis revealed that EC extract possessed high levels of carbohydrates (74.77 ± 1.15%) and ash (19.66 ± 0.16%), followed by proteins (2.88 ± 0.05%), moisture (1.44 ± 0.48%), and fat (1.26 ± 0.18%). The level of 3,6-anhydro-D-galacose was roughly 43.74 ± 6.30 μmol/L.

### Effect of EC extract on cell viability

**Fig 2:**
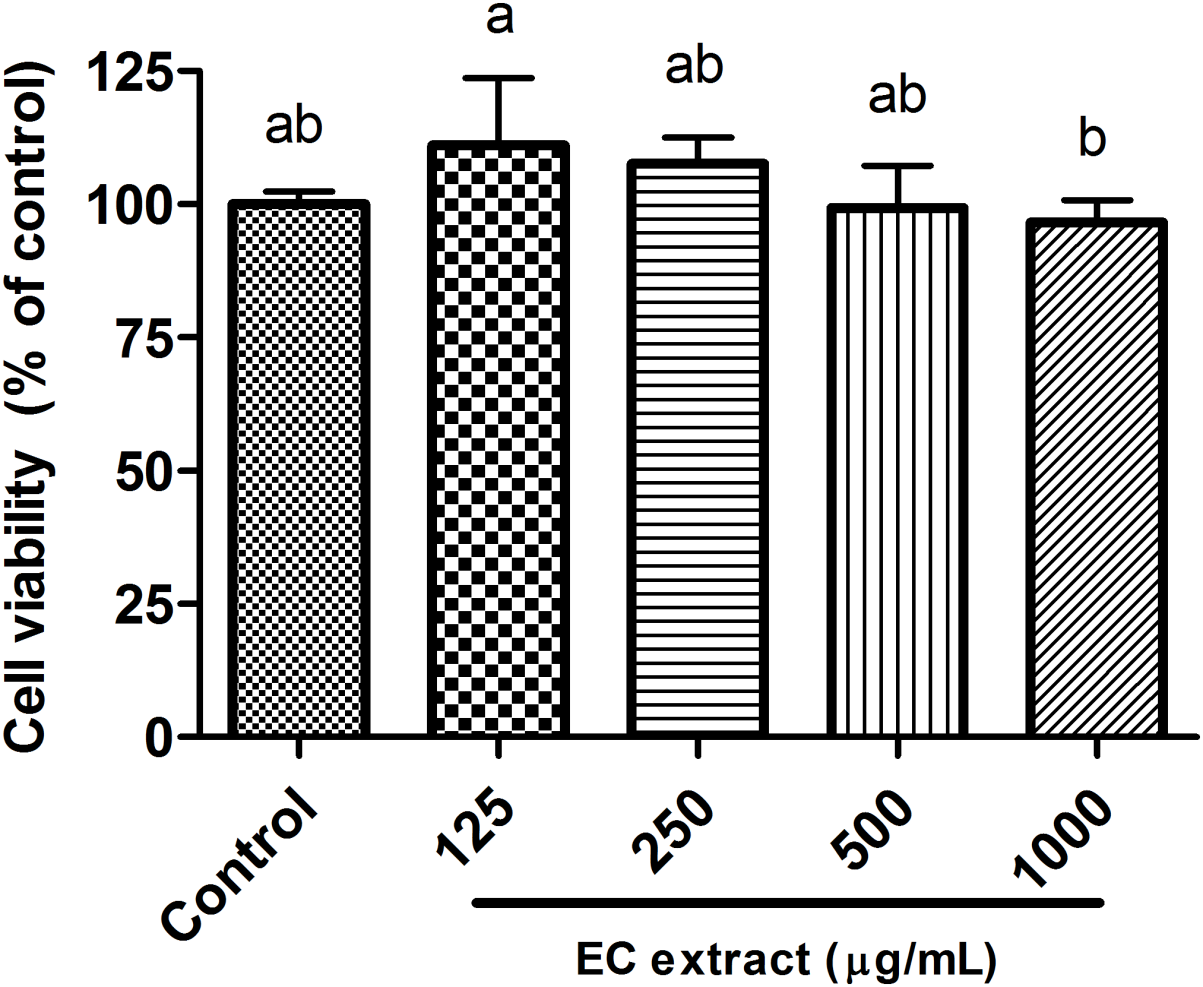
Cell viability of RAW 264.7 macrophage after treatment with EC extract for 24 h. Data are shown as the mean ± S.D. (n = 5). Different letters (a-b) indicate statistical significance (P<0.05) as determined by Duncan’s multiple range test.

EC extract was not toxic to RAW 264.7 macrophage cells (Fig 2) or normal cells. Cell viability between EC extract- and control-treated cells did not differ significantly. Seaweed extracts have been previously reported to be non-toxic to normal cells [47] and human keratinocyte (HaCaT) cells [26].

### Weight loss and disease activity index scores

**Fig 3:**
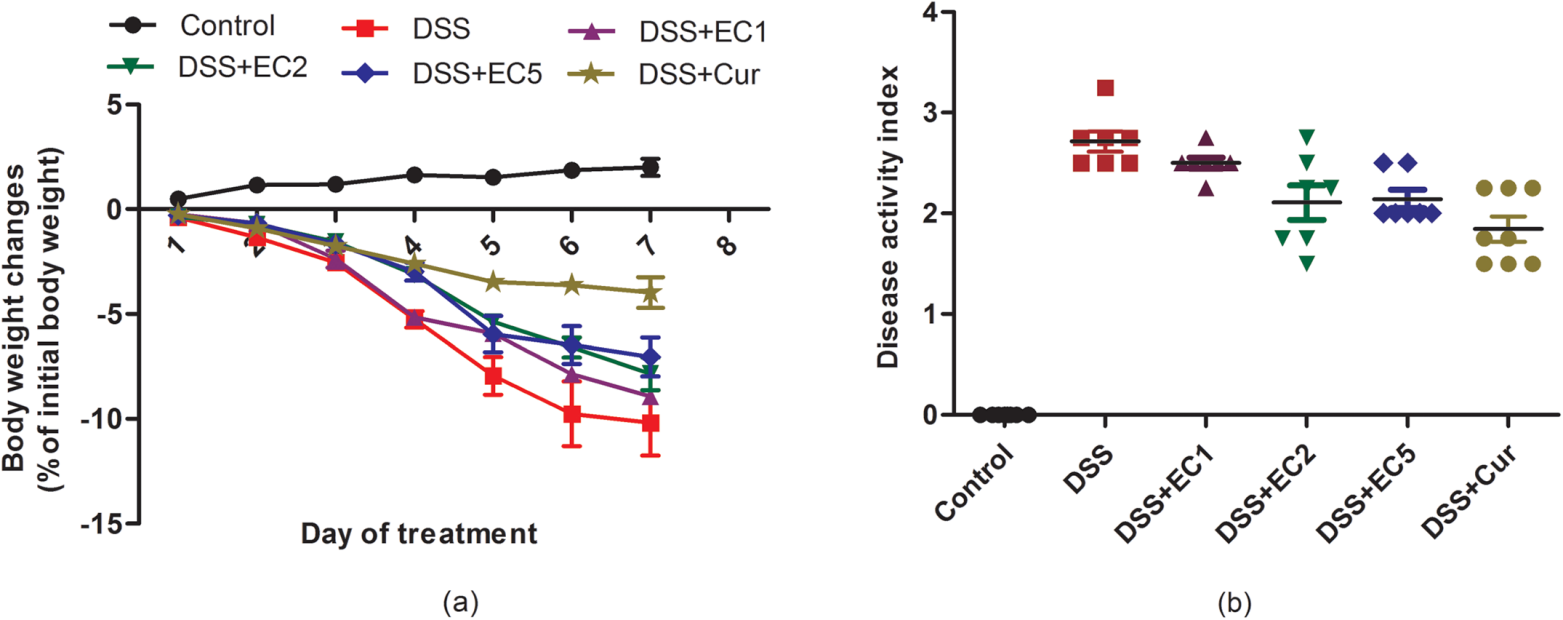
Effect of EC extract on (a) weight loss and (b) disease activity index in DSS-treated mice during and after 7 days of treatment. Data are shown as the mean ± S.D. (n = 8). Letters indicate statistical significance (P<0.05) as analyzed by Duncan’s multiple range test.

Body weight loss was increased in DSS-treated mice, with EC extract or Curcumin administration attenuating body weight loss extent (Fig 3a). Disease activity index scores were higher in untreated DSS-treated mice versus EC- and Curcumin-treated counterparts (Fig 3b).

### Colon weight and length

**Fig 4:**
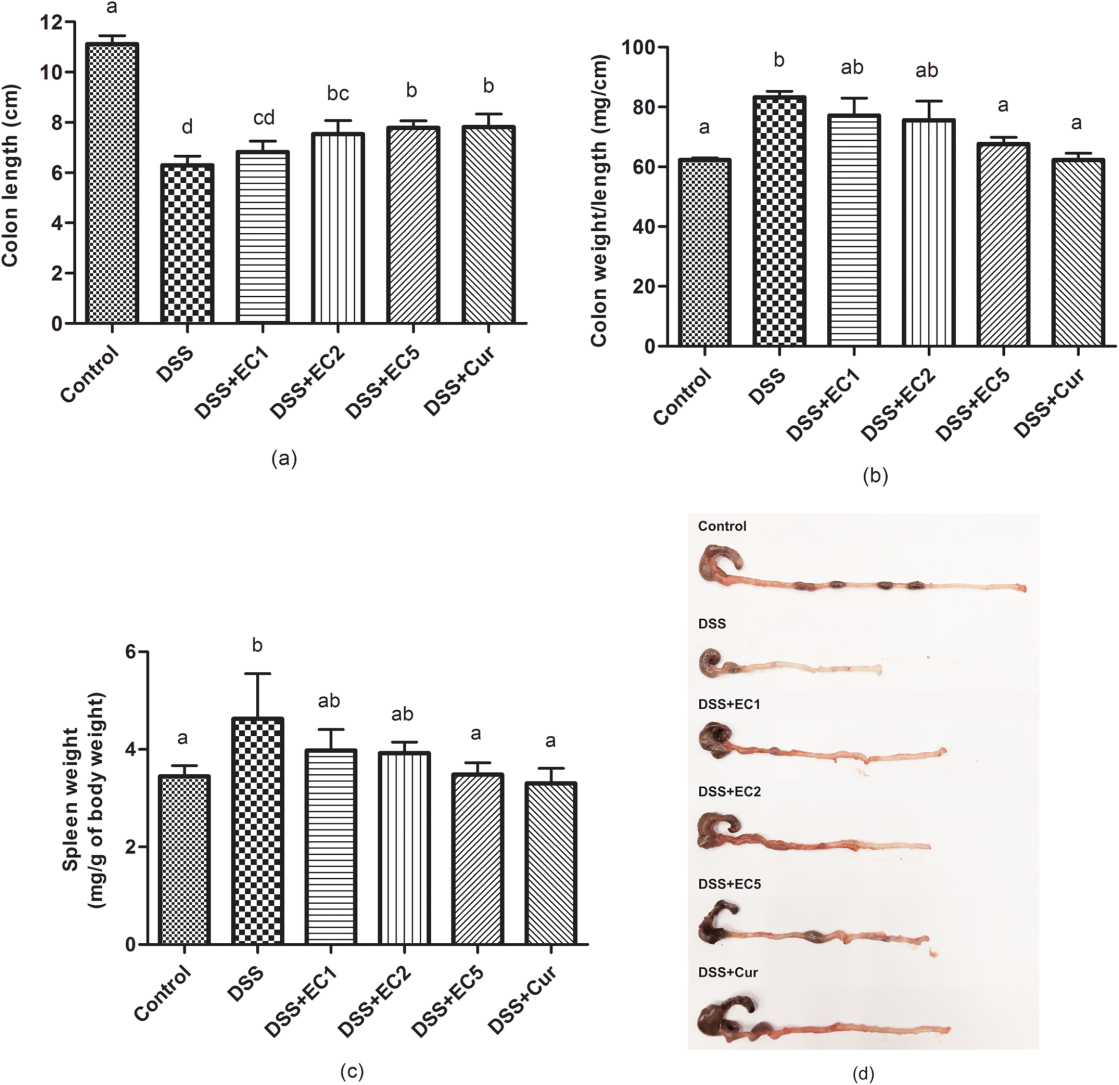
Effects of EC extract treatment on colon health after 7 days of treatment: (a) colon length, (b) colon weight/length ratio, (c) splenic weight, (d) representative colons for each group. Data are shown as the mean ± S.D. (n = 8). Letters indicate statistical significance (P<0.05) as analyzed by Duncan’s multiple range test.

Colon length was decreased in DSS-treated mice after 7 days of treatment. Administration of a high dosage of EC extract or Curcumin significantly (P<0.05) attenuated colon shortening (Fig 4a). In addition, both treatments also resulted in decreased colon weight per length ratio (Fig 4b) and reduced splenic weight (Fig 4c). Fig 4d shows a representative colon for each group.

### Inflammatory cytokines

**Fig 5:**
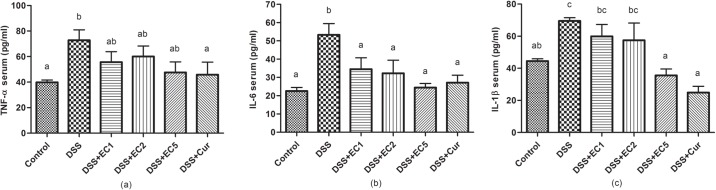
Effects of EC extract on inflammatory cytokine expression in serum after 7 days of treatment: (a) TNF-α, (b) IL-6, and (c) IL-1β. Data are shown as the mean ± S.D. (n = 8). Letters indicate statistical significance (P<0.05) as analyzed by Duncan’s multiple range test.

**Fig 6:**
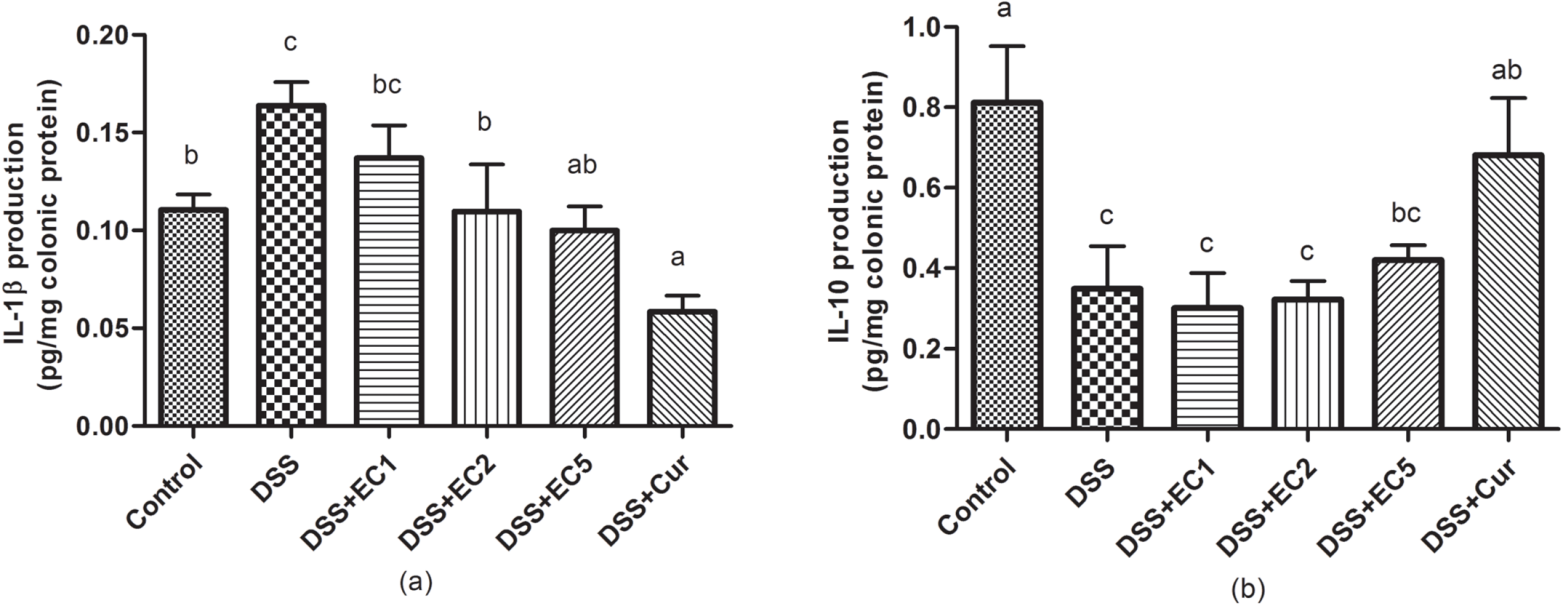
Effects of EC extract on inflammatory cytokine expression in colon tissue after 7 days of treatment: (a) IL-1β and (b) IL-10. Data are shown as the mean ± S.D. (n = 8). Letters indicate statistical significance (P<0.05) as analyzed by Duncan’s multiple range test.

After DSS treatment, pro-inflammatory cytokine levels (e.g., TNF-α, IL-1β, and IL-6) were significantly (P<0.05) increased relative to control mice (Fig 5a and 5b). Treatment with EC extract or Curcumin reduced TNF-α, IL-1β, and IL-6 levels in serum. In addition, EC extracts also significantly (P<0.05) reduced IL-1β expression in colon tissues (Fig 6a). IL-10 expression was high in healthy mice and decreased in DSS-treated mice. Treatment with EC extract or Curcumin both regulated IL-10 expression in colon tissue (Fig 6b).

### Colonic histopathology

**Fig 7:**
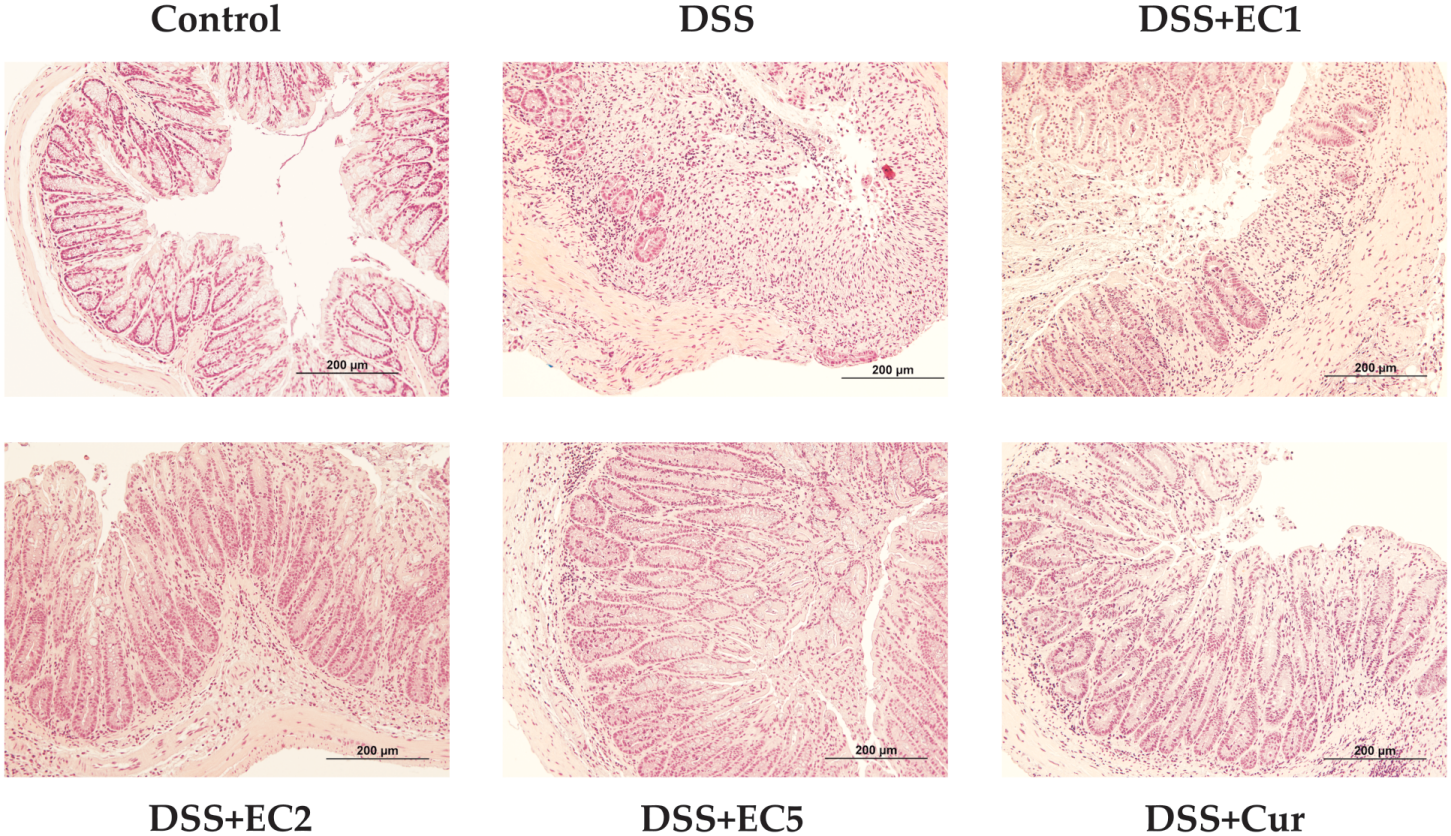
Representative colonic tissue histopathology for each group after 7 days of treatment.

Under hematoxylin and eosin (H&E) observation, the normal colon shows good wall layer architecture and no loss of crypt cells (Score 0). However, DSS-treated mice presented thicker mucosal layers accompanied by erosion (Score 4, Fig 7). After treatment with EC extract (especially high doses) or Curcumin, crypt cell loss was ameliorated (Score 1).

## Discussion

Inflammatory bowel diseases, including Crohn’s disease and ulcerative colitis, can be recognized by intestinal inflammation [34]. Various animal models have been developed for studying IBD pathogenesis, including dextran sodium sulfate (DSS), trinitrobenzene sulfonic acid (TNBS), oxazolone, acetic acid, carrageenan, indomethacin (a non-steroidal anti-inflammatory drug [NSAID]), and peptidoglycan-polysaccharide induced models [32]. DSS administration in the drinking water is a commonly used method for inducing colitis in mice owing to its simplicity, reproducibility, controllability, and rapidity. DSS dosages given to BALB/c mice have ranged between 2.5–5.0% [34].

Body weight decreased in mice with DSS-induced colitis, with EC extract or Curcumin administration both helping to attenuate this body weight loss (Fig 3a). In addition, the disease activity index scores were also increased in mice with colitis (Fig 3b). These results are in accordance with previous studies [34, 35, 45]. Colitis induced by DSS was associated with acute histological changes such as weight loss, diarrhea, and rectal bleeding, resulting in increased disease activity index scores. The oral administration of either EC extract or Curcumin alleviated disease activity index scores in DSS-treated mice. Colitis was also characterized by colon shortening (Fig 4a and 4c) and increased colon weight/length ratios (Fig 4b), with EC extract or Curcumin treatment preventing this increase in colon weight. Overall, treatment with EC extract or Curcumin ameliorated the clinical signs and symptoms of colitis induced in mice by DSS.

Molecular observations supported the beneficial effects of EC extract and Curcumin, as treatment with either reduced the levels of pro-inflammatory cytokines such as TNF-α, IL-1β, and IL-6 in DSS-treated mouse sera. In contrast, untreated DSS-mice showed increased expression levels of these cytokines (Fig 5). TNF-α, IL-6, and IFN-γ are the main inflammatory mediators in murine colitis models [35, 48]. TNF signaling has been found to induce the pleiotropic pro-inflammatory effects of colitis, including the activation of effector T cells and macrophages. TNF signaling also directed intestine epithelial cell (ICE) damage via myosin light chain kinase (MLCK) activation [49, 50]. Increased IL-6 production by the lamina propria and CD4^+^ T cells has been reported in experimental colitis models [51]. A recent study reported that the administration of anti-TNF-α may be a strategy for managing colitis [52]. Colitis can also be prevented by regulating IL-10 expression [49, 53]. The present study showed that in DSS-treated mice, IL-10 expression in colon tissues was lower than in animals treated with EC extract or Curcumin (Fig 6b). A previous study has also reported that untreated IL-10 gene-deficient mice showed progressive histopathological injury, increased colon weight/length ratios, and elevated IFN-γ and IL-17 expression [54]. Colitis severity is related to colonic damage severity. Here, untreated mice presented thicker mucosal layers and erosion. EC extract or Curcumin treatment both reduced histopathological score severity and resulted in a high number of crypt cells in the mucosal layer (Fig 7). A previous study has reported that mice treated with 5% DSS presented mucosal thickness and epithelial injury as well as increased microscopic damage scores [45].

Red seaweeds are mostly composed of sulfated galactans such as carrageenan and agar [15], which are made up of repeating disaccharide units of alternating 3-linked *β*-D-galactopyranose and 4-linked *α*-D-galactopyranose or 4-linked 3,6-anhydro-*α*-D-galactopyranose. These can be extracted from red seaweeds (Rhodophyta) such as *Eucheuma*, *Hypnea*, *Gigartina*, and *Chondrus crispus* [55, 56]. Dietary fibers include polysaccharides, oligosaccharides, lignins, and other compounds associated with plant substances. Dietary fiber, as edible parts of plants or analogous carbohydrates, are resistant to digestion and absorption in the human small intestine, resulting in complete or partial fermentation in the large intestine [57]. A recent review reported that dietary fermented oligosaccharides, disaccharides, monosaccharides, and polyols (FODMAP) possessed beneficial effects for IBD patients by reducing levels of the pro-inflammatory markers C-reactive protein and fecal calprotein [58].

The EC extract has shown antioxidant, anti-inflammation, and anti-cancer properties [26, 27, 59, 60], and has previously been demonstrated to slow tumor cell growth rate [27], promote wound healing [28], and upregulate cancer cell apoptosis [29]. Moreover, extracts of this seaweed improved cardiovascular, liver, and metabolic parameters in obese rat models [30] and presented anti-diabetic effects in streptozotocin-induced type 2 diabetic mice [31].

Used in this study as a positive control, Curcumin has been demonstrated to prevent colitis by suppressing NF-κB [61] and inhibiting STAT3 signaling [62]. In addition, Curcumin can also modulate certain inflammatory mediators such as TNF-α and nitric oxide [63]. Future work should focus on microbiota variation in murine intestines, as previous studies have reported that the gut microbiota is altered in cases of IBD [64, 65].

## Conclusions

Here, a *Eucheuma cottonii* ethanol (EC) extract has demonstrated suppressive effects on colonic disease induced by DSS in mice. EC extract treatment reduced weight loss and disease activity index scores, as well as regulating the levels of pro-inflammatory cytokines such as TNF-α, IL-6, and IL-1β. EC extract administration also reduced colon injury in DSS-treated mice. As such, the dietary polysaccharides found in the *E*. *cottonii* extract may be used for the future treatment of colitis.
